# Epigenome-wide association study of posttraumatic stress disorder identifies novel loci in U.S. military veterans

**DOI:** 10.1038/s41398-022-01822-3

**Published:** 2022-02-17

**Authors:** Janitza L. Montalvo-Ortiz, Joel Gelernter, Zhongshan Cheng, Matthew J. Girgenti, Ke Xu, Xinyu Zhang, Shyamalika Gopalan, Hang Zhou, Ronald S. Duman, Steven M. Southwick, John H. Krystal, Traumatic Stress Brain Research Study Group, Matthew J. Friedman, Ronald S. Duman, Matthew J. Girgenti, John H. Krystal, Janitza L. Montalvo-Ortiz, Robert H. Pietrzak

**Affiliations:** 1 Department of Psychiatry, Yale University School of Medicine, New Haven, CT USA; 2 VA CT Healthcare Center, West Haven, CT USA; 3 National Center for PTSD, West Haven, CT USA; 4 Center for Genetic Epidemiology, Keck School of Medicine, University of Southern California, Los Angeles, CA USA; 5 Department of Psychiatry, Yale University School of Medicine, New Haven, CT USA; 6 VA CT Healthcare Center, West Haven, CT USA; 7 National Center for PTSD, West Haven, CT USA; 8 Department of Psychiatry, Geisel School of Medicine at Dartmouth, Hanover, USA

**Keywords:** Clinical genetics, Prognostic markers

## Abstract

Posttraumatic stress disorder (PTSD) is a chronic and disabling psychiatric disorder prevalent in military veterans. Epigenetic mechanisms have been implicated in the etiology of PTSD, with DNA methylation being the most studied to identify novel molecular biomarkers associated with this disorder. We performed one of the largest single-sample epigenome-wide association studies (EWAS) of PTSD to date. Our sample included 1135 male European–American U.S. veterans who participated in the National Health and Resilience in Veterans Study (NHRVS). DNA was collected from saliva samples and the Illumina HumanMethylation EPIC BeadChip was used for the methylation analysis. PTSD was assessed using the PTSD Checklist. An EWAS was conducted using linear regression adjusted for age, cell-type proportions, first 10 principal components, and smoking status. After Bonferroni correction, we identified six genome-wide significant (GWS) CpG sites associated with past-month PTSD and three CpGs with lifetime PTSD (*p*_range_ = 10^−10^–10^−8^). These CpG sites map to genes involved in immune function, transcription regulation, axonal guidance, cell signaling, and protein binding. Among these, *SENP7*, which is involved in transcription regulation and has been linked to risk-taking behavior and alcohol consumption in genome-wide association studies, replicated in an independent veteran cohort and was downregulated in medial orbitofrontal cortex of PTSD postmortem brain tissue. These findings suggest potential epigenetic biomarkers of PTSD that may help inform the pathophysiology of this disorder in veterans and other trauma-affected populations.

## Introduction

Posttraumatic stress disorder (PTSD) is one of the most prevalent psychiatric disorders in veterans and the disorder most closely associated with military experience. PTSD is a chronic and often disabling condition characterized by intrusive symptoms, avoidance, negative cognitions and mood, and alterations in arousal and reactivity. The estimated lifetime prevalence of PTSD in the general population of U.S. veterans is 6.9% [1] and among combat-exposed veterans is as high as 20% [2].

Epigenetic mechanisms may be implicated in the long-term effects of traumatic stress. DNA methylation, the most studied epigenetic modification in humans, plays an important role in the effects of trauma-related events as a risk factor for psychiatric disorders [3–5]. There have been several studies examining the epigenetic contribution to PTSD risk at the genome-wide level. A burgeoning body of epigenome-wide association studies (EWAS) examining differentially methylated CpG sites in PTSD have been conducted, with the most promising findings implicating the immune system in peripheral tissue [6–8]. However, most of these studies were underpowered and did not adjust for differences in cell-type proportions when studying peripheral tissue, as well as other factors such as technical batch effects or smoking status, which may confound purported associations between these epigenetic markers and PTSD.

An EWAS of PTSD in 96 Australian male Vietnam War veterans found that methylation sites in *BRSK1*, *LCN8*, *NFG*, and *DOCK2* genes were significantly associated with the severity of PTSD symptoms [9]. Findings for the *DOCK2* gene, which is implicated in Alzheimer’s disease pathology [10], were replicated in an independent sample of 115 African Americans (AAs) from the Grady Trauma Project [9]. Gene enrichment analysis revealed actin cytoskeleton and focal adhesion pathways associated with PTSD. An EWAS study using a larger sample of 473 World Trade Center responders did not identify any genome-wide significant (GWS) methylation sites associated with PTSD. However, enrichment analysis identified several pathways associated with top differentially methylated CpG sites that included synaptic plasticity, oxytocin plasticity, cholinergic synapse, and inflammatory disease pathways [11]. A meta-EWAS study of PTSD in civilian and military cohorts (*n* = 1147 subjects from seven different cohorts) from the PGC-PTSD consortium Epigenetics workgroup also did not identify GWS associations for PTSD [12], possibly due to the large heterogeneity among study cohorts and type of trauma. A meta-EWAS study of three civilian cohorts (*n* = 545 subjects) identified two CpG sites significantly associated with current-PTSD mapping to the *NRG1* and *HGS* genes [13]. A recent study in 513 trauma-exposed veterans of the post-9/11 conflicts showed one GWS finding at *G0S2* gene, which was replicated in the military cohort from the Consortium PTSD EWAS [14]. Another recent EWAS in 1896 PTSD cases and trauma-exposed controls identified significant differential methylation at 4 CpG sites mapping to *AHRR*, with supportive evidence from metabolomics data [15]. A summary of these studies is included in Table 1. However, most of these findings from these studies have not been yet replicated in independent datasets and/or have been conducted using a lower resolution array for DNA methylation. Larger samples using arrays with better DNA methylation coverage are needed to identify novel loci associated with PTSD.

**Table 1:** Cross-sectional epigenome-wide association studies of PTSD in peripheral tissue.

Reference	Sample Size (*N*)	Study design	Tissue	Array	Main findings
Uddin et al. [6]	100	23 lifetime PTSD	Peripheral Blood	HumanMethylation27 BeadChip	Genes involved in the immune function were un-methylated among PTSD individuals.
Smith et al. [7]	110	50 PTSD	Peripheral Blood	HumanMethylation27 BeadChip	PTSD is associated with increased global methylation and differential methylation in genes involved in immune function.
Mehta et al. [8]	169	61 PTSD	Peripheral Blood	HumanMethylation450K BeadChip	PTSD cases with child abuse show distinct and almost nonoverlapping gene expression and DNA methylation profiles.
Ratanatharathorn et al. [12]	1174	587 PTSD	Peripheral Blood	HumanMethylation450K BeadChip	No genome-wide significant findings. PGC-PTSD Epigenetics QC pipeline was introduced.
Mehta et al. [9]	96	48 current PTSD	Peripheral Blood	HumanMethylationEPIC BeadChip	*DOCK2* was significantly associated with PTSD and replicated in an independent sample (n = 115). Pathways identified include actin cytoskeleton and focal adhesion.
Hammamieh et al. [46]	159	71 PTSD	Peripheral Blood	Agilent Whole-genome DNA Methylation array (~27 K) HumanMethylation450K BeadChip	Unknown genome-wide significant findings; 3339 differentially methylated genes. Pathways enriched include somatic complications, endocrine signaling, nervous system development and function.
Kuan et al. [11]	473	171 current PTSD	Peripheral Blood	HumanMethylation450K BeadChip	No genome-wide significant findings. Major depressive disorder was also assessed.
Uddin et al. [13]	545	196 current PTSD	Peripheral Blood	HumanMethylation450K BeadChip	*NRG1* and *HGS* were significantly associated with PTSD.
Logue et al. [14]	513	378 lifetime PTSD	Peripheral Blood	HumanMethylationEPIC BeadChip	*G0S2* was significantly associated with PTSD. Some evidence of association with *AHRR*.
Smith et al. [15]	1,896	796 PTSD	Peripheral Blood	HumanMethylation450K BeadChip	Decreased *AHRR* methylation at four CpG sites. Supported by metabolomics findings.

In this study, which is one of the largest to date with a comparatively homogenous sample, we examined epigenome-wide DNA methylation signatures of PTSD in a large, nationally representative cohort (*n* = 1135) of male European–American (EA) U.S. veterans using a high-density array. We conducted an EWAS of both current (past-month) and lifetime PTSD showing replication findings in an independent cohort of veterans and post-EWAS analyses, including symptom-level sensitivity analysis and methylation quantitative trait analysis. We also evaluated the gene expression of our top finding in postmortem brain tissue of an independent sample of individuals with PTSD.

## Methods and materials

### Study cohort

Our sample included a total of 1,135 EA male U.S. military veterans who participated in the National Health and Resilience in Veterans Study (NHRVS) [16, 17], a nationally representative study of U.S. veterans, conducted in October-December 2011. Participants were recruited from the Knowledge Networks research panel comprising more than 50,000 households that are developed and maintained by Ipsos, a survey research firm. All participants provided informed consent. This study was approved by the Human Subjects Subcommittee of the Veterans Affairs (VA) Connecticut Healthcare System and VA Office of Research & Development. Table 2 illustrates the demographic and clinical characteristics of the sample.

**Table 2:** Demographic and clinical characteristics of the European–American male veteran study cohort (*n* = 1,135).

	Total Current PTSD		Lifetime PTSD	
	Cases	Controls	Cases	Controls
*N*_total_	1135			
No. (%)	34 (3.0)	1,101 (97)	65 (5.7)	1,070 (94.3)
Age, Mean (SD), *y*	62 (12.2)			
	60 (11.8)	66 (12.1)	61 (10.9)	66 (12.1)
Current smoker, No. (%)	137 (12.1)			
	10 (29.4)	127 (11.5)	21 (32.3)	116 (10.8)
Alcohol use disorder, No. (%)	532 (46.8)			
	29 (78.4)	503 (45.8)	56 (87.5)	476 (44.4)
Tobacco use disorder, No. (%)	235 (20.7)			
	11 (29.7)	224 (20.4)	26 (40.6)	209 (19.5)
Substance use disorder, No. (%)	167 (14.7)			
	14 (37.8)	153 (13.9)	31 (48.4)	136 (12.7)

### PTSD assessment

PTSD was assessed using the PTSD Checklist-Specific (PCL-S), a 17-item self-report measure that assesses DSM-IV symptoms of PTSD related to each respondent’s worst traumatic event assessed using the Trauma History Screen [18]. This assessment allows for individual-level screening for PTSD, aiding in diagnostic assessment of PTSD, and monitoring change in PTSD symptoms. A total symptom severity score was calculated by summing the scores from the 17 items (score range for each item = 1–5); our scores range from 17–85. We also computed symptom cluster scores using a five-factor model of PTSD symptoms that includes re-experiencing, avoidance, emotional numbing, dysphoric arousal (e.g., sleep disturbance), and anxious arousal (e.g., hypervigilance) [19, 20]. Both lifetime and current (i.e., past-month) PTSD symptoms were assessed, with scores ≥ 50 indicative of a positive screen for PTSD [21], which is conservative for general population samples [22]. The median current and lifetime PTSD PCL scores were 20 ± 9.3 (Cronbach’s α = 0.94, and 23 ± 11.2 (Cronbach’s α = 0.94), respectively. A total of 65 (5.7%) veterans met this conservative screening cut-off for lifetime PTSD and 43 (3.0%) for current PTSD. Prevalence for alcohol and drug use disorder, which were assessed using modified self-report version of the MINI Neuropsychiatric Interview [23]; and tobacco use disorder, which was assessed using the Fagerström Test for Nicotine Dependence [24], are also shown in Table 2. Information about the most common index traumas in each group is included in the Supplementary Material.

### DNA extraction and preparation for epigenetic analysis

Genomic DNA (500 ng) was extracted from saliva samples using Oragene kits (DNA Genotek, Ottawa, Ontario, Canada) and treated with bisulfite reagents included in the EZ-96 DNA methylation kit (Zymo Research, Orange, CA, USA) following the manufacturer’s standard protocol. Bisulfite-converted DNA samples were then assessed with the Illumina Infinium Human MethylationEPIC BeadChip (Illumina, San Diego, CA, USA), which interrogates DNA methylation of >850,000 loci across the genome at single-nucleotide resolution.

### Microarray processing and quality control

Genome-wide DNA methylation assay was conducted at the Yale Center for Genome Analysis (YCGA). Quality control was conducted in R version 3.4.1 and performed based on a recently published pipeline using the ‘minfi’ R package (Bioconductor 1.8.9) [25]. Quality control and normalization are described in the Supplementary Material. After quality control and normalization, a total of 706,573 CpG sites (82% of sites) were left for subsequent analysis.

Since methylation values at CpG sites can be cell-type specific [26], we conducted a cell composition estimation analysis using a modified version of the Housemann method [27, 28]. The relative proportion of each cell type (e.g., CD34, CD14, and buccal cells) was estimated in our heterogeneous peripheral saliva samples, and included in the model. To adjust for possible population stratification (within EAs), a methylation-based principal component (PC) approach [29] was conducted based on sets of CpG sites within 50 kb of SNPs using the 1000 Genomes Project variants with minor allele frequency (MAF) > 0.1 following the Barfield et al. method [29]. The first 10 PCs were included in the model as covariates to adjust for population stratification.

### Gene ontology analysis

A gene ontology analysis was conducted using the “gometh” function in the Bioconductor R package missMethyl [30]. This function accounts for the varying number of CpG sites per gene by providing a prior probability for each gene based on gene length, followed by a modified hypergeometric test for overrepresentation of a gene set [31]. We evaluated possible overrepresentation in GO and KEGG pathways among the CpG sites at a false discovery rate (FDR) < 0.05 for current and lifetime PTSD. The FDR was set at 0.05 to adjust for multiple testing.

### Methylation quantitative trait loci

Methylation quantitative trait locus (meQTL) analysis was conducted to evaluate the influence of genotype variation on CpG methylation patterns. Single-nucleotide polymorphisms (SNPs) within ±50 kb of the CpG site (hg19 reference genome) were included in the meQTL analysis. Genotyping information, imputation, and quality control methods are included in the Supplementary Material. PLINK 1.9 [32] was used for the linear regression analysis with SNPs located ±50 kb from each GWS CpG site with its corresponding beta value. Covariates included in the linear regression model of the meQTL analysis were age, smoking status, cell type proportion, and PCs. A Bonferroni correction was used to adjust for multiple testing of 12,475 SNPs that were within ±50 kb of the 9 GWS CpG sites (0.05/12,475 = 4.0 × 10^−6^).

### Replication analysis

We conducted a replication analysis in 608 male veterans from the Veterans Aging Cohort Study (VACS), a nationwide multicenter collaborative project designed to understand the role of co-morbid medical and psychiatric diseases on HIV infection (https://medicine.yale.edu/intmed/vacs/). The VACS biobank is comprised of 2,470 participants who were recruited for genetic studies from 2006 to 2007. Participants in the VACS biobank provided informed consent for the genetic study and provided blood samples. Clinical and demographic data were collected within 90-days of the blood sample collection.

Diagnosis of PTSD was derived from the electronic medical record. A total of 112 veterans were diagnosed with PTSD, and 496 veterans without PTSD served as controls. All veterans were men with mean age 49.4 ± 7.6 years old and a majority were African Americans (86%) and HIV-positive. Genomic DNA was extracted from whole blood samples. DNA methylation profiling, quality control, normalization and data analysis are described in Supplementary Material. DNA methylation was assessed using a lower coverage array, the Illumina HumanMethylation 450 K array. Three out of the nine GWS CpG sites were not tested because they are not included in the higher density array used in the discovery dataset.

### Postmortem Brain PTSD Analysis for top signal mapped to *SENP7*

Given the limited postmortem brain tissue available, we prioritized the replicated *SENP7* findings for further examination and validation. Human autopsy brain tissue samples were obtained from the National Center for PTSD Brain Bank and the University of Pittsburgh Medical Center. Fresh frozen samples of prefrontal cortex from medial orbitofrontal cortex (Broadmann area 11, BA11) were collected from each postmortem sample. This brain region was selected as it is an integral hub of the limbic system and plays an important role in PTSD by modulating inhibitory and regulatory functions, among other processes [33]. Quantitative real-time PCR (qRT-PCR) was performed on dissected tissue from 18 subjects with PTSD (mean age, 46.9 ± 12.3 years, 8 females) and 17 matched neurotypical controls (mean age, 48.1 ± 12.7 years, 9 females) of European ancestry. The average postmortem interval (PMI) was 17.1 h in the PTSD cohort and 19.3 h for the controls. There were no significant differences between the PTSD and control samples in age, postmortem interval, pH, or RNA integrity number. Causes of death was similar between PTSD and control samples. In the PTSD sample, the most common cause of death was “natural” (61%), followed by “accidental” (28%). 2 PTSD cases had “suicide” as cause of death. In the control sample, the most common cause of death was also “natural” (76%), followed by “accidental” (24%). None of the control samples have “suicide” as cause of death. qRT-PCR was performed using primers designed to detect the transcript of *SENP7* (See **Results** and Supplementary Material). Fold regulation was calculated by using the –delta delta Ct (2-DDCt) method.

### Statistical Analysis

Epigenome-wide analysis study (EWAS) analyses were conducted using the ‘cpg.assoc’ function from the ‘minfi’ R package in which we conducted a series of linear regressions (for current and lifetime PTSD) using age, cell type proportion (CD34, CD14, buccal), first 10 principal components, and smoking status as covariates. Given the heteroscedasticity of beta values, M-values (logit transformation in log2 scale) were calculated and used in all analyses, as recommended by Du et al. [34]. Bonferroni correction was used to adjust for multiple testing (0.05/706,573; *p* = 7.07 ×10^−8^). For the sensitivity analysis of PTSD symptoms, a linear regression was conducted using the PTSD symptoms cluster as the outcome, and M value and covariates in the model. For the sensitivity analysis for substance use disorders in our SENP7*cg09657378 finding, we conducted a multi-variable regression analysis, using M-values of SENP7*cg09657378 as the dependent variable and lifetime PTSD, covariates, and either substance use or substance use disorder variables as independent variables (See Supplementary Material).

For the postmortem gene expression analysis, differences in transcriptional changes between PTSD and neurotypical controls were evaluated using Graphpad Prism v7 (Graphpad Software, San Diego, CA) with fold changes calculated using the 2-DDCt method. Mann–Whitney U test followed by Bonferroni correction for multiple comparisons was used to assess statistical differences in fold regulation.

## Results

### Epigenome-wide association analysis

**Fig. 1:**
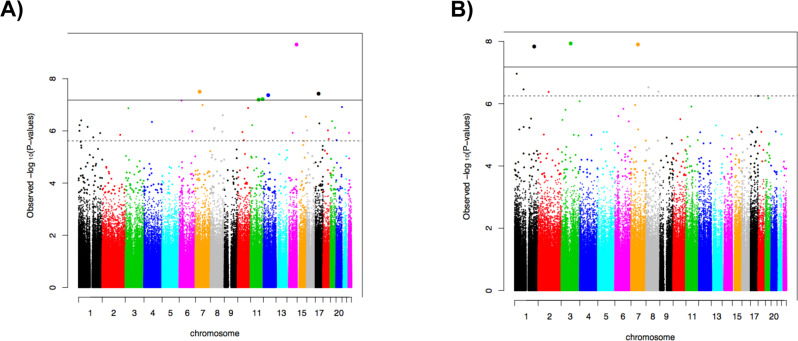
Manhattan plots of the association between DNA methylation and PTSD. **A** Current PTSD, **B** Lifetime PTSD. The horizontal solid line represents Bonferroni-corrected significance. The dotted line indicates false discovery rate of < 0.05.

Quantile-quantile plots are shown in Supplementary Fig. 1, with lambda values of 1.006 and 1.020 for current and lifetime PTSD, respectively. After Bonferroni correction, a total of 6 GWS CpG sites were associated with current PTSD (Table 3A). The Manhattan plot is depicted in Fig. 1A. Our top CpG site, cg07672479 (chr14: 102431106, *p* = 5.49 × 10^−10^) maps to Dynein Cytoplasmic 1 Heavy Chain 1 (*DYNC1H1*). Other GWS CpG sites differentially methylated with respect to current PTSD included cg03284870 (chr7:45197346, *p* = 3.47 × 10^−8^), cg22500183 (chr17:33914271, *p* = 4.09 × 10^−8^), cg06595994 (chr12:51632641, *p* = 4.68 × 10^−8^), and cg00770699 (chr11:85370479, *p* = 7.02 × 10^−8^) located within the *RAMP3* (Receptor Activity Modifying Protein 3), *AP2B1 (*Adaptor Related Protein Complex 2 Subunit Beta 1), *DAZAP2 (*DAZ Associated Protein 2), and *CREBZF (*CREB/ATF BZIP Transcription Factor) genes, respectively. An additional intergenic GWS CpG site associated with current PTSD was cg15559076 (chr11:128109597, *p* = 6.60 × 10^−8^). Hypomethylation at five GWS CpG sites (all except cg00770699) was associated with current PTSD, whereas hypermethylation at one site, cg00770699, was associated with current PTSD (Supplementary Fig. 2A).

**Table 3:** Genome-wide significant DNA methylation sites associated with PTSD in U.S. veterans.

A. Current PTSD					
CpG Site ID	Chromosome	Location	Gene symbol	Gene name	*P*-Value
*(A) Current PTSD*					
cg07672479	14	102431106	*DYNC1H1*	Dynein Cytoplasmic 1 Heavy Chain 1	5.49 × 10^−10^
cg03284870	7	45197346	*RAMP3*	Receptor Activity Modifying Protein 3	3.47 × 10^−8^
cg22500183	17	33914271	*AP2B1*	Adaptor Related Protein Complex 2 Subunit Beta 1	4.09 × 10^−8^
cg06595994	12	51632641	*DAZAP2*	DAZ Associated Protein 2	4.68 × 10^−8^
cg15559076	11	128109597			6.60 × 10^−8^
cg00770699	11	85370479	*CREBZF*	CREB/ATF BZIP Transcription Factor	7.02 × 10^−8^
*(B) Lifetime PTSD*					
cg09657378	3	101232109	*SENP7*	SUMO Specific Peptidase 7	1.71 × 10^−8^
cg07377876	7	73868114	*GTF2IRD1*	GTF2I Repeat Domain Containing 1	1.81 × 10^−8^
cg19825186	1	207495562	*CD55*	CD55 molecule	2.08 × 10^−8^

Functional annotation of the current-PTSD-associated GWS CpG sites showed that one CpG site (cg07672479; *DYNC1H1*) is promoter-associated, one is located in the 5′UTR region (cg06595994, *DAZAP2*), and one in the gene body (cg00770699; *CREBZF*). Further, cg06595994 is located at an enhancer. A total of five GWS CpG sites (all except cg00770699) have three Dnase hypersensitivity sites, an indicator of open chromatin, whereas cg00770699 has six transcription factor binding sites.

In the lifetime PTSD EWAS analysis, we identified a total of three Bonferroni-corrected GWS CpG sites (Table 3B). The Manhattan plot is depicted in Fig. 1B. These GWS CpG sites included cg09657378 (chr3:101232109, *p* = 1.71 × 10^−8^), cg07377876 (chr7:73868114, *p* = 1.81 × 10^−8^), and cg19825186 (chr1:207495562, *p* = 2.08 × 10^−8^); they map to *SENP7* (SUMO Specific Peptidase 7), *GTF2IRD1* (GTF2I Repeat Domain Containing 1), and *CD55* (CD55 molecule) genes, respectively. Hypomethylation in all three GWS CpG sites was associated with lifetime PTSD (Supplementary Fig. 2B). Functional annotation of these CpG sites showed that two are promoter-associated (cg09657378 and cg19825186), and one is located in the 5′UTR region (cg19825186). Further, cg07377876 is enhancer related and all three CpG sites have three Dnase hypersensitivity sites.

### Sensitivity analysis

**Fig. 2:**
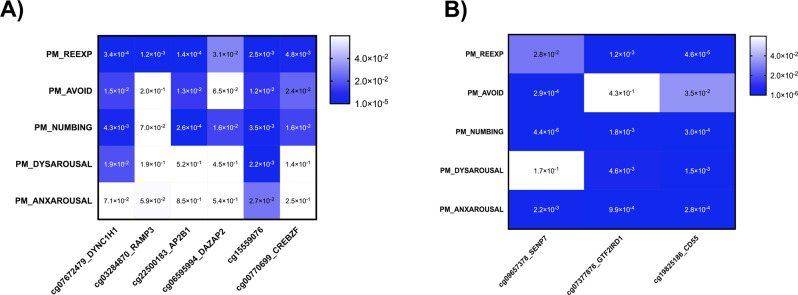
Heatmap of *P*-values significance for genome-wide significant (GWS) CpG sites and PTSD symptomatology. **A** Current PTSD, **B** Lifetime PTSD. Heatmap colors correspond to the significance (*p*-value), where darker indicates higher degree of association, lighter indicates a lower degree of association, and white indicates no association.

To evaluate the relationship between the DNA methylation of GWS CpG sites identified and PTSD symptom clusters (i.e., re-experiencing, avoidance, emotional numbing, dysphoric arousal, anxious arousal) [19, 20], we conducted linear regression analyses using the same covariates as in the EWAS analysis (Fig. 2). For current PTSD, all GWS CpG sites were significantly associated with re-experiencing, with cg07672479 showed the strongest association (*p* = 3.4 × 10^−04^). A total of four GWS CpG sites were significantly associated with avoidance (cg07672479, cg22500183, cg1559076, and cg00770699), and five with emotional numbing (all except for cg03284870). Only two GWS CpG sites were significantly associated with dysphoric arousal (cg07672479 and cg15559076), and one with anxious arousal (cg15559076).

For lifetime PTSD, all GWS CpG sites were significantly associated with re-experiencing, emotional numbing, and anxious arousal. The strongest associations were cg19825186 for re-experiencing (*p* = 4.6 × 10^−05^), cg09657378 for emotional numbing (*p* = 4.4 × 10^−06^), and cg19825186 for anxious arousal (*p* = 2.8 × 10^−04^). For avoidance, all but cg07377876 were significantly associated, with cg09657378 showing the strongest association (*p* = 2.9 × 10^−04^). For dysphoric arousal, all but cg09657378 were significantly associated, with the strongest association for cg19825186 (*p* = 1.5 × 10^−03^).

### Gene ontology analysis

For lifetime PTSD, the KEGG pathway ‘Viral myocarditis’ was identified (*p* = 2.45 × 10^−06,^ FDR = 8.15 × 10^−04^; Supplementary Table 1). For current PTSD, no KEGG pathways were identified. No GO ontologies were enriched at FDR 0.05 for either current or lifetime PTSD.

### meQTL analysis for top methylation probes

A total of 30 meQTLs were identified (*p*-value range: 10^−11^–10^−6^) (Supplementary Fig. 3). These mapped to cg15559076, which is nearby the *EST1* gene. The most significant meQTL for cg15559076 was at chr11:128025697 (rs10893823; beta = −0.28, *p* = 7.14 × 10^−11^).

### Replication analysis

For the replication analysis, a total of 608 male veterans (112 PTSD cases, and 496 controls) from the VACS biobank cohort were evaluated. Methylation levels at the *SENP7* CpG site (cg09657378, promoter region) were associated (*p* = 0.02) with PTSD diagnosis. Hypomethylation at cg09657378 was observed in PTSD cases, showing the same directional effect as in the discovery dataset.

### Differential expression of *SENP7* in Postmortem PTSD Brain Tissue

**Fig. 3:**
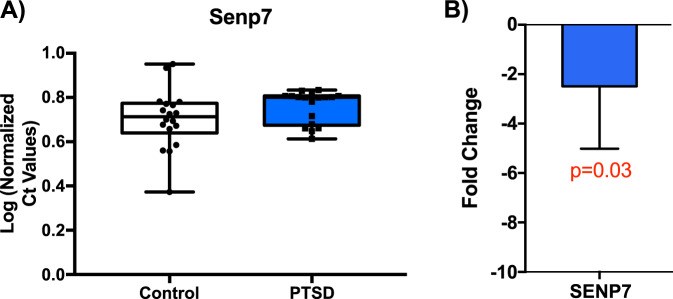
Postmortem Brain PTSD Findings in Medial Orbitofrontal Cortex. Based on the significant observed association and then the replication of the *SENP* (cg09657378) association in the VACS cohort, we examined gene expression in postmortem brain tissue from medial orbitofrontal cortex (Broadmann 11) in PTSD cases and controls. **A** Box plots of normalized Ct values for *SENP7* in controls (white), and PTSD (blue) individuals. **B** Log 2-fold change of *SENP7* in human mOFC (*p* < 0.03, *n* = 35). Error bars indicate ± SEM.

Postmortem brain tissue from mOFC (Broadmann 11) from subjects with PTSD matched to neurotypical controls of European ancestry (*n* = 17–18/group) were assayed for gene expression changes by quantitative real-time PCR. Normalized log corrected Ct values were used to calculate fold change. This analysis revealed a significant 1.4 fold decrease in *SENP7* expression (*P* = 0.03; Fig. 3A, B, error bars indicate ± SEM).

## Discussion

To date, EWAS of PTSD have been limited in successfully identifying associated loci, most likely due to lack of power, high heterogeneity of the cohorts studied, and failure to replicate in independent cohorts. In this study, we conducted the largest to date EWAS of PTSD in a single population of U.S. military veterans, and identified novel differentially methylated CpG sites associated with both current and with lifetime PTSD. Specifically, we identified nine GWS CpG associated with PTSD: six were associated with current (past-month) PTSD and three with lifetime PTSD. Of these, a CpG site mapping to *SENP7* was replicated in a demographically and clinically distinct cohort of veterans. Follow-up validation in an independent sample of postmortem brain tissue from individuals with PTSD and matched controls showed downregulation of *SENP7* in the mOFC.

Our top finding in the EWAS of current PTSD is cg07672479, which is annotated to the *DYNC1H1* gene. This gene encodes for cytoplasmic dynein 1, which plays a role in retrograde axonal transport, neuronal migration, and immune function, and is highly expressed in the brain (https://www.proteinatlas.org/ENSG00000197102-DYNC1H1/tissue). Mutations in *DYNC1H1* are associated with Charcot-Marie-Tooth disease [35], which is characterized by progressive weakness and atrophy, spinal muscular atrophy [36], and severe intellectual disability [37].

The second GWS CpG site associated with current PTSD included cg03284870, which is located in *RAMP3*, a gene involved in GPCR signaling; and the third, cg22500183, maps to the *AP2B1* gene, which encodes the adaptor protein complex 2, which is implicated in synaptic vesicle pathway, axonal guidance, and immune function. This gene is highly expressed in the brain, endocrine tissues, and male tissues (https://www.protein atlas.org/ENSG00000006125-AP2B1/tissue). Additional GWS CpG sites associated with current PTSD include cg06595994 located in the *DAZAP2* gene, which encodes for a protein involved in cell signaling, transcription regulation, and spermatogenesis, cg15559076 located in an intergenic region, and cg00770699 which maps to *CREBZF* involved in MAPK-Erk pathway, which is implicated in the long-term formation of fear-related memories [38].

For lifetime PTSD, three GWS CpG sites were identified: cg09657378, annotated to the *SENP7* gene; cg073777876, annotated to the *GTF2IRD1*; and cg19825186 annotated to the *CD55* gene. *SENP7* encodes for an enzyme that deconjugates SUMO2 and SUMO3, which are involved in transcription regulation. Of note, this gene has been previously identified in genome-wide association studies (GWAS) of risk-taking behavior (*n* = 315,894) and alcohol consumption (*n* = 414,343) [39]. The second gene, *GTF2IRD1*, is also implicated in transcription regulation and has been identified in GWAS of neuroticism (*n* = 329,821) [40]. Lastly, *CD55* is a gene involved in the innate immune response, specifically in the complement cascade. Gene enrichment analysis of GWS CpG sites of lifetime PTSD at FDR < 0.05 identified the “viral myocarditis” KEGG pathway, implicating immune function and cardiovascular disorders.

Half of the GWS CpG sites for current PTSD are only present in the high-density DNA methylation array (cg06595994, cg15559076, and cg00770699), showing that use of this array increased ability to identify significant CpG sites associated with current PTSD. To determine whether changes in methylation at these CpG sites are influenced by genotype, we integrated the genotype data and identified 30 meQTLs (*p*-value range: 10^−11^–10^−6^) mapping to cg15559076. The closest-mapped gene is *ETS1*, implicated in immune regulation. SNPs at this gene have been also identified in GWAS of celiac disease [41].

When comparing EWAS findings of current and lifetime PTSD, we found some overlap (Supplementary Table 2). All GWS CpG sites from the lifetime PTSD EWAS showed suggestive association in current-PTSD EWAS (*p*-value range: 10^−5^–10^−4^). In the current-PTSD EWAS, 4 out of 6 GWAS CpG sites showed significance in the lifetime PTSD EWAS (*p*-value range: 10^−6^–10^−3^). The other two CpG sites (cg22500183, *AP2B1*; cg06595994, *DAZAP2*) did not show any nominal p-value in the lifetime PTSD EWAS. This suggests that differential methylation at these sites may not persist over time.

For replication, we examined our GWS hits an independent veteran cohort of European- and African-American ancestry. We observed a significant replication of the association of *SENP7* gene methylation (cg09657378, *p* = 0.02) with PTSD diagnosis, with the same direction effect of hypomethylation of *SENP7* associated with PTSD. Of note, this association was independent of substance use or substance use disorder comorbidity in the discovery cohort (See Supplementary Table 3). Evaluating postmortem brain from the mOFC of an independent cohort of individuals with PTSD and matched controls, we found evidence of downregulation of *SENP7* in PTSD. cg09657378 is located in the promoter region. While it is generally expected that hypomethylation in the promoter region is associated with gene activation, recent studies have found that its effects may be gene-specific, leading to either increased or decreased gene expression [42]. Future studies should evaluate whether differential methylation directly influences *SENP7* gene expression and examine its effects on the behavioral level.

Overall, results of the current study are consistent with previous EWAS studies of PTSD implicating differential methylation of genes implicated in the immune system. Our results also revealed novel genes involved in axon guidance, transcription regulation, and cell signaling. They further suggest that associations between DNA methylation of these CpG sites and PTSD may be driven by specific PTSD symptomatology. Specifically, for current PTSD, all GWS CpG sites were associated with re-experiencing (i.e., intrusive thoughts, nightmares, and flashbacks of the traumatic event), and are considered the most characteristic set of PTSD symptoms. However, for dysphoric and anxious arousal symptoms, the association is present in cg15559076, an intergenic region but with enhancer activity, and cg07672479, which is annotated to *DYNC1H1*, which is implicated in retrograde axonal transport, neuronal migration, and immune function, and is highly expressed in the brain. These PTSD symptom clusters, which are characterized by sleep disturbance, concentration difficulties, and hypervigilance, are common to many anxiety-related disorders and are not specific to PTSD. In lifetime PTSD, all GWS CpG sites are associated with re-experiencing, but also numbing and dysphoric arousal symptoms. Differential methylation at cg09657379 and cg19825186 were observed in relation to avoidance symptoms, whereas cg07377876 and cg19825186 in dysphoric arousal. Notably, one might speculate of an apparent distinction between current and lifetime PTSD: while current PTSD is associated with genes implicated in neuroplasticity (i.e., response to stress), lifetime PTSD is associated with traits related to poor impulse control (i.e., the likelihood of exposing oneself to stressful situations). However, further studies are needed to evaluate the specificity of these associations and the extent to which epigenetic markers reflect the causes or consequences of PTSD symptoms.

To the best of our knowledge, this is the largest EWAS of PTSD to date that evaluated epigenetic markers associated with both current and lifetime PTSD in a veteran cohort. Study strengths are the investigation of a relatively large and homogeneous cohort, the use of a high-density DNA methylation array, evaluation of both current and lifetime PTSD as well as PTSD symptom clusters; integration of genotype data; replication in an independent veteran cohort; and postmortem brain validation of the top replicated EWAS hit.

To confirm our findings, replication in independent well-powered samples and in more heterogeneous samples in terms of sex and race, are warranted. This study is further limited by the lack of gene expression data in the discovery cohort, and the use of saliva, a peripheral tissue, for DNA methylation profiling since DNA methylation patterns are tissue-specific [20]. Nevertheless, several studies comparing the DNA methylation patterns across different peripheral tissues and the brain consistently show the highest correlation between saliva and brain [28, 43, 44]. However, the changes observed in peripheral tissue may not necessarily reflect those in the brain. Further, PTSD not only impacts the brain, but also the numerous other bodily systems. Examination of epigenetic changes in peripheral tissue may thus be informative in identifying biological pathways implicated in PTSD other than the brain, for example, the immune system. Accumulating evidence in peripheral tissue from previous EWAS of PTSD and other studies show alterations in the immune function [6–8]. Our findings suggest that these changes could occur system-wide, but may also be gene-level specific as has been shown previously [45]. Further, if personalized epigenetics results are ever to have treatment implications, they need to derive from accessible tissues such as blood or saliva, despite the limitations of this approach.

## Conclusions

We identified nine GWS DNA methylation sites associated with current or lifetime PTSD in U.S. military veterans—six for current PTSD and three for lifetime PTSD. These mapped to genes involved in immune function, axon guidance, transcription regulation, and cell signaling pathways, and showed differential associations with PTSD symptom clusters. Further, alterations of *SENP7* in PTSD were replicated in an independent cohort of veterans and validated in postmortem brain analysis. Future research should focus on integrating brain and peripheral methylomics and transcriptomics datasets, together with GWAS data, to identify molecular biomarkers of PTSD. Our findings provide new insights into the epigenetic architecture underlying the complex etiology of PTSD.

## Supplementary information

Supplementary material

## Data Availability

Code will be available upon request.
