# Supplementary material for: Epigenome-wide association study of posttraumatic stress disorder identifies novel loci in U.S. military veterans
Source: Transl Psychiatry. 2022 Feb 17;12:65. doi: 10.1038/s41398-022-01822-3 (PMC8854688; doi:10.1038/s41398-022-01822-3)
Supplement: Supplementary file 1 — Supplementary material [file 41398_2022_1822_MOESM1_ESM.docx]

Epigenome-Wide Association Study of Posttraumatic Stress Disorder

Identifies Novel Loci in U.S. Military Veterans

Janitza L. Montalvo-Ortiz^1,2,3^, Joel Gelernter^1,2,3^, Zhongshan Cheng^1^, Matthew J. Girgenti^1,2,3^, Ke Xu^1,2,3^, Xinyu Zhang^1,2^, Shyamalika Gopalan^4^, Hang Zhou^1,2^, Ronald S. Duman^1,2,3†^ Steven M. Southwick^1,2,3^, John H. Krystal^1,2,3^, Traumatic Stress Brain Research Study Group*, Robert H. Pietrzak^1,2,3^

*Author Affiliations:*

^1^Division of Human Genetics, Department of Psychiatry, Yale University School of Medicine, New Haven, CT; ^2^VA CT Healthcare Center, West Haven, CT; ^3^National Center for PTSD; ^4^Department of Ecology and Evolution, Stony Brook University, Stony Brook, NY.

^†^Deceased 1 February 2020. *A list of contributing authors and their affiliations appears at the end of the paper.

*Corresponding Author:*

Janitza L. Montalvo-Ortiz, PhD. Yale University School of Medicine, Department of Psychiatry, VA Connecticut Healthcare System, 950 Campbell Avenue; West Haven, CT 06516; email: [janitza.montalvo-ortiz@yale.edu](mailto:joel.gelernter@yale.edu)

*Short title:* Epigenomics of PTSD in Veterans

**Supplementary Material**

**Study cohort**

The most common index trauma in the NHRVS cohort was ‘sudden death of close family member or friend’ (n=307, 27%). In the PTSD cases, the most common index trauma was ‘during military service – saw something horrible or was badly scared’ (n=10, 29% for current PTSD, and n=17, 26% for lifetime PTSD), whereas in controls, ‘sudden death of close family member or friend’ (n=301, 27% for current PTSD, and n=290, 27% for lifetime PTSD).

All NHRVS subjects were genotyped using the Illumina PsychArray. Subjects with missing genotype call rate > 0.05, mismatched sex information, excessed heterozygosity rate, duplicated or with more than 7 relatives in the sample were removed. Single nucleotide polymorphisms (SNPs) with genotype call rate <= 0.05, minor allele frequency (MAF) < 0.01, or Hardy-Weinberg Equilibrium (HWE) p-value < 1e-6 were removed. Principle component analysis (PCA)^1^ was applied for population stratification using 1000 Genome (1000G) phase3^2^ as reference panel to classify European-Americans.

**Microarray processing and quality control**

To ensure that only high-confidence probes were included, the following were filtered out: 1) probes with detection *p*-value > 0.001, 2) probes with annotated single nucleotide polymorphisms (SNPs) at SBE/CpG sites (via the Single Nucleotide Polymorphism database 137 [National Center for Biotechnology Information), 3) cross-hybridizing probes which mapped to multiple places in the genome, and 4) sex chromosomes probes. Combat method in the ‘sva’ package^3^ was applied to correct for batch effects due to cohort group and sample plate. DNA methylation data were normalized using functional normalization in the ‘minfi’ R package,^4^, which uses internal control probes present on the array to control for between-array technical variation. This method reportedly outperforms other existing approaches.^5^

**Replication analysis**

***Profiling DNA methylation using Illumina DNA methylation Beadchips***

Genomic DNA was extracted from whole blood samples. DNA methylation profiling was conducted at the Yale Center for Genomic Analysis using the Illumina (San Diego, CA, USA) Infinium HumanMethylation450 BeadChip (HM450K). All samples were randomly placed on each array and batch-corrected using the removeBatchEffect function in limma.^6^ Probe normalization and batch-correction was performed as previously described by Lehne *et al*.^7^

***Data quality control and normalization***

A total of 11,648 probes on sex chromosomes and 36,142 probes within 10 base pairs of single nucleotide polymorphisms were removed. 437,722 probes remained for analysis. As described by Lehne et al^7^, 24,416 probes on Y chromosomes were applied to evaluate the detection p-value. A p<1E-12 was set as a detection p value threshold to improve the quantification of methylation intensities. The intensity values with detection *p*>1E-12 were labeled missing, and samples with a call rate < 98% were excluded. We also compared the predicted sex with self-reported sex. All samples matched as male. Six cell types (CD4+ T cells, CD8+ T cells, NK T cells, B cells, monocytes, and granulocytes) in the blood were estimated in each sample using the method described by Houseman^8^. The first Principal Component Analysis (PCA) was performed to evaluate the intensity values of positive control probes designed in HM450. Then, the first GLM was performed as follows: Methylation ~ Age + race + alcohol + antiretroviral therapy + log_10_VL + WBC + CD8 Tcell + CD4 Tcell + Granulocyte + NK + B cell + Monocyte + PC_ControlProbe_1-30. The residuals for each probe and the top 30 PCs of the first PCA were used to adjust for technical biases, particularly batch effects. The second PCA was performed on the resulting regression residuals from the first model. The top 5 PCs of the second PCA were used to control for global biological confounders that cannot be directly captured in the model.

The final GLM model was performed as: Methylation ~ PTSD + Age + race + alcohol + medication adherence + log_10_VL+ WBC + CD8 Tcell + CD4 Tcell + Gran + NK + B cell + Mono + PC_ControlProbe_1-30 + PC_Residual_1-5.

**Postmortem Brain PTSD Analysis for top signal mapped to *SENP7***

Quantitative real-time PCR (qRT-PCR) was performed on dissected tissue from Broadmann Area 11, the medial orbitofrontral cortex (mOFC) in 18 subjects with PTSD (mean age, 46.9 +/- 12.3 years, 8 females) and 17 matched neurotypical controls (mean age, 48.1 +/- 12.7 years, 9 females) of European ancestry. The average postmortem interval (PMI) was 17.1 hours in the PTSD cohort and 19.3 hours for the controls. There were no significant differences between the PTSD and control samples in age, PMI, pH, or RNA integrity number. qRT-PCR was performed using primers designed to detect the transcripts of *SENP7*. mRNA was isolated from the mOFC using the RNEasy Plus Mini Kit (Qiagen, Venlo, Netherlands); 1 ug of mRNA was reverse-transcribed into cDNA using the iScript cDNA Synthesis kit (Bio-Rad, Hercules, CA). RNA was hydrolyzed and resuspended in nuclease free water. Gene specific primers for *SENP7* (GTCCTGGGAACGGAGTTAGG, TGAAGAAAGTTGCTCTGATTGC), and the control gene *GAPDH* (ACCCAGAAGACTGTGGATGG, GAGGCAGGGATGATGTTCTG) were designed using Primer 3 v.0.4.0 freeware (<http://bioinfo.ut.ee/primer3-0.4.0/)> and tested for efficiency and specificity by serial dilution and melt curve analysis. Sybr Green mix (Bio-Rad, Hercules, CA) was used to amplify cDNA.

**Sensitivity analysis**

To evaluate the effect of SUDs on PTSD-associated differential DNA methylation and considering the limited power in our EWAS, we performed a SUD sensitivity analysis using a targeted approach. We evaluated the effect of substance use and SUDs in our replicated genome-wide significant finding, *SENP7**cg09657378. We conducted a multi-variable regression analysis using the following models:

**Substance use model:**

M values of *SENP7**cg09657378 ~ LT PTSD + Age + CD34 + CD14 + Buccal + PC1 + PC2 + PC3 + PC4 + PC5 + PC6 + PC7 + PC8 + PC9 + PC10 + Smoking + Alcohol use + Cannabis use + Cocaine use + Stimulants use + Narcotics use + Hallucinogens use + Phencyclidine use + Inhalants use + Tranquilizers use.

**Substance use disorder model**

M values of *SENP7**cg09657378 ~ LT PTSD + Age + CD34 + CD14 + Buccal + PC1 + PC2 + PC3 + PC4 + PC5 + PC6 + PC7 + PC8 + PC9 + PC10 + Substance use disorder + Alcohol use disorder +

Tobacco use disorder.

**Consortia authorship**

Traumatic Stress Brain Research Group

Matthew J. Friedman ^3,4^ (Director), Victor E. Alvarez ^3,5,6^, David Benedek ^7^, Christopher Brady ^3,5,6^, Dianne Cruz ^8^, David A Davis ^9^, Ronald S. Duman ^1,2,3†^, Matthew J. Girgenti ^1,2,3^, Paul E. Holtzheimer ^3,4^, Bertrand R. D. Huber ^3,5,6^, Terence M. Keane ^3,5,6^, Neil Kowell ^5,6^, John H. Krystal ^1,2,3^, Mark W. Logue ^3,5,6^, Ann McKee ^5,6^, Brian Marx ^3,5,6^, Deborah Mash ^10^, Mark W. Miller ^3,5,6^, Janitza L. Montalvo-Ortiz ^1,2,3^, William K. Scott ^9^, Thor Stein ^5,6^, PhD, Robert Ursano ^7^, Douglas E. Williamson ^8,11^, Erika J. Wolf ^3,5,6^, Keith A. Young ^12^

^1^ Division of Human Genetics, Department of Psychiatry, Yale University School of Medicine, New Haven, CT, ^2^ VA CT Healthcare Center, West Haven, CT, ^3^ National Center for PTSD, ^4^ Department of Psychiatry, Geisel School of Medicine at Dartmouth, ^5^ Departments of Psychiatry, Neurology, Biomedical Genetics, Biostatistics and/or Pathology & Laboratory Science, Boston University, ^6^ VA Boston Healthcare System, ^7^ Department of Psychiatry, Uniformed Services University of Health Sciences, ^8^ Department of Psychiatry & Behavioral Sciences, Duke University Medical Center, ^9^ Departments of Human Genetics, Public Health Sciences and/or Neurology, University of Miami Miller School of Medicine, ^10^ Department of Biomedical Sciences, Nova Southeastern University, ^11^ Durham VA Healthcare System, ^12^ Department of Psychiatry & Behavioral Science, Texas A&M University System Health Science Center. ^†^Deceased 1 February 2020.

**References:**

1 Galinsky, K. J., Bhatia, G., Loh, P. R., Georgiev, S., Mukherjee, S., Patterson, N. J. et al. Fast Principal-Component Analysis Reveals Convergent Evolution of ADH1B in Europe and East Asia. American journal of human genetics, 2016;98:456-472. doi:10.1016/j.ajhg.2015.12.022.

2 Genomes Project, C., Auton, A., Brooks, L. D., Durbin, R. M., Garrison, E. P., Kang, H. M. et al. A global reference for human genetic variation. Nature, 2015;526:68-74. doi:10.1038/nature15393.

3 Leek, J. T., Johnson, W. E., Parker, H. S., Jaffe, A. E. ,Storey, J. D. The sva package for removing batch effects and other unwanted variation in high-throughput experiments. Bioinformatics, 2012;28:882-883. doi:10.1093/bioinformatics/bts034.

4 Aryee, M. J., Jaffe, A. E., Corrada-Bravo, H., Ladd-Acosta, C., Feinberg, A. P., Hansen, K. D. et al. Minfi: a flexible and comprehensive Bioconductor package for the analysis of Infinium DNA methylation microarrays. Bioinformatics, 2014;30:1363-1369. doi:10.1093/bioinformatics/btu049.

5 Fortin, J. P., Labbe, A., Lemire, M., Zanke, B. W., Hudson, T. J., Fertig, E. J. et al. Functional normalization of 450k methylation array data improves replication in large cancer studies. Genome biology, 2014;15:503. doi:10.1186/s13059-014-0503-2.

6 Smyth, G. K. in *Bioinformatics and Computational Biology Solutions Using R and Bioconductor* (eds Robert Gentleman *et al.*) 397-420 (Springer New York, 2005).

7 Lehne, B., Drong, A. W., Loh, M., Zhang, W., Scott, W. R., Tan, S. T. et al. A coherent approach for analysis of the Illumina HumanMethylation450 BeadChip improves data quality and performance in epigenome-wide association studies. Genome biology, 2015;16:37. doi:10.1186/s13059-015-0600-x.

8 Houseman, E. A., Kelsey, K. T., Wiencke, J. K. ,Marsit, C. J. Cell-composition effects in the analysis of DNA methylation array data: a mathematical perspective. BMC Bioinformatics, 2015;16:95. doi:10.1186/s12859-015-0527-y.

**Supplementary Table 1.**

**A. Current PTSD**

| **KEGG** | **N** | **DE** | **P.DE** | **FDR** |
| --- | --- | --- | --- | --- |
| Insulin secretion | 83 | 2 | 0.00135 | 0.45 |
| Vasopressin-regulated water reabsorption | 43 | 2 | 0.00356 | 0.59 |
| Endocrine and other factor-regulated calcium reabsorption | 49 | 2 | 0.00688 | 0.76 |
| Synaptic vesicle cycle | 78 | 2 | 0.01541 | 1 |
| Dilated cardiomyopathy (DCM) | 92 | 2 | 0.02457 | 1 |
| Fluid shear stress and atherosclerosis | 137 | 2 | 0.02905 | 1 |
| Vascular smooth muscle contraction | 130 | 2 | 0.03319 | 1 |
| Platelet activation | 123 | 2 | 0.03620 | 1 |
| Apelin signaling pathway | 136 | 2 | 0.04185 | 1 |
| Mismatch repair | 22 | 1 | 0.04195 | 1 |
| **GO** | **N** | **DE** | **P.DE** | **FDR** |
| Secretory vesicle | 944 | 8 | 0.00024 | 1 |
| Regulation of response to wounding | 156 | 4 | 0.00024 | 1 |
| Negative regulation of response to wounding | 83 | 3 | 0.00068 | 1 |
| Tolerance induction | 24 | 2 | 0.00075 | 1 |
| Endomembrane system | 4166 | 17 | 0.00133 | 1 |
| Asparagine biosynthetic process | 2 | 1 | 0.00183 | 1 |
| Asparagine synthase (glutamine-hydrolyzing) activity | 2 | 1 | 0.00183 | 1 |
| Regulation of wound healing | 132 | 3 | 0.00210 | 1 |
| Adrenomedullin receptor complex | 3 | 1 | 0.00211 | 1 |
| Adrenomedullin receptor activity | 3 | 1 | 0.00211 | 1 |

**B. Lifetime PTSD**

| **KEGG** | **N** | **DE** | **P.DE** | **FDR** |
| --- | --- | --- | --- | --- |
| Viral myocarditis | 55 | 3 | 2.45E-06 | 8.15E-04 |
| Glutathione metabolism | 53 | 1 | 0.01958 | 0.89 |
| Ferroptosis | 37 | 1 | 0.01961 | 0.89 |
| Basal transcription factors | 41 | 1 | 0.02110 | 1 |
| Cysteine and methionine metabolism | 47 | 1 | 0.02418 | 1 |
| Complement and coagulation cascades | 76 | 1 | 0.02428 | 1 |
| Pathogenic Escherichia coli infection | 54 | 1 | 0.02568 | 1 |
| Vibrio cholera infection | 49 | 1 | 0.02736 | 1 |
| Hematopoietic cell lineage | 90 | 1 | 0.03195 | 1 |
| Salmonella infection | 80 | 1 | 0.03430 | 1 |
| **GO** | **N** | **DE** | **P.DE** | **FDR** |
| Regulation of glutamate-cysteine ligase activity | 1 | 1 | 0.00062 | 1 |
| Positive regulation of glutamate-cysteine ligase activity | 1 | 1 | 0.00062 | 1 |
| Glutamate-cysteine ligase catalytic subunit binding | 1 | 1 | 0.00062 | 1 |
| T cell mediated immunity | 97 | 2 | 0.00066 | 1 |
| Regulation of memory T cell activation | 1 | 1 | 0.00083 | 1 |
| Positive regulation of memory T cell activation | 1 | 1 | 0.00083 | 1 |
| MHC class Ib protein complex binding | 2 | 1 | 0.00095 | 1 |
| MHC class Ib protein binding | 2 | 1 | 0.00095 | 1 |
| Transition between slow and fast fiber | 1 | 1 | 0.00104 | 1 |
| Cellular response to thyroxine stimulus | 2 | 1 | 0.00123 | 1 |

**Supplementary Table 2.**

**A. Current PTSD**

| **CpG Site ID** | **Chromosome** | **Location** | **Gene Symbol** | **Current PTSD**  ***P* Value** | **Lifetime PTSD**  ***P* Value** |
| --- | --- | --- | --- | --- | --- |
| cg07672479 | 14 | 102431106 | *DYNC1H1* | 5.49 × 10^-10^ | 3.36 × 10^-05^ |
| cg03284870 | 7 | 45197346 | *RAMP3* | 3.47 × 10^-08^ | 1.47 × 10^-06^ |
| cg22500183 | 17 | 33914271 | *AP2B1* | 4.09 × 10^-08^ | 3.78 × 10^-01^ |
| cg06595994 | 12 | 51632641 | *DAZAP2* | 4.68 × 10^-08^ | 1.37 × 10^-01^ |
| cg15559076 | 11 | 128109597 |  | 6.60 × 10^-08^ | 1.81 × 10^-04^ |
| cg00770699 | 11 | 85370479 | *CREBZF* | 7.02 × 10^-08^ | 4.06 × 10^-03^ |

**B. Lifetime PTSD**

| **CpG Site ID** | **Chromosome** | **Location** | **Gene Symbol** | **Lifetime PTSD**  ***P* Value** | **Current PTSD**  ***P* Value** |
| --- | --- | --- | --- | --- | --- |
| cg09657378 | 3 | 101232109 | *SENP7* | 1.71 × 10^-08^ | 5.75 × 10^-05^ |
| cg07377876 | 7 | 73868114 | *GTF2IRD1* | 1.81 × 10^-08^ | 1.61 × 10^-04^ |
| cg19825186 | 1 | 207495562 | *CD55* | 2.08 × 10^-08^ | 8.78 × 10^-04^ |

**Supplementary Table 3.**

1. **The effect of substance use on the association of *SENP7**cg09657378 and lifetime PTSD**

| **Variable** | **Estimate** | **Standard error** | **95% CI (asymptotic)** | **\|t\|** | **P value** | **P value summary** |
| --- | --- | --- | --- | --- | --- | --- |
| Intercept | -2.926 | 0.6926 | -4.285 to -1.567 | 4.224 | <0.0001 | **** |
| LT PTSD | -0.1445 | 0.02708 | -0.1977 to -0.09140 | 5.337 | <0.0001 | **** |
| Age | -0.002405 | 0.0005808 | -0.003544 to -0.001265 | 4.141 | <0.0001 | **** |
| Smoking | 0.03581 | 0.01982 | -0.003078 to 0.07470 | 1.807 | 0.0711 | ns |
| CD34 | -2.671 | 0.5894 | -3.828 to -1.515 | 4.532 | <0.0001 | **** |
| CD14 | -1.635 | 0.4709 | -2.559 to -0.7111 | 3.472 | 0.0005 | *** |
| Buccal | -2.214 | 0.5485 | -3.291 to -1.138 | 4.037 | <0.0001 | **** |
| Comp.1 | 13.05 | 12.87 | -12.21 to 38.31 | 1.013 | 0.3110 | ns |
| Comp.2 | -3.711 | 1.151 | -5.970 to -1.452 | 3.223 | 0.0013 | ** |
| Comp.3 | -0.1935 | 0.2408 | -0.6659 to 0.2789 | 0.8038 | 0.4217 | ns |
| Comp.4 | -0.03837 | 0.2693 | -0.5668 to 0.4900 | 0.1425 | 0.8867 | ns |
| Comp.5 | 0.3833 | 0.2114 | -0.03141 to 0.7980 | 1.813 | 0.0700 | ns |
| Comp.6 | -0.1155 | 0.2073 | -0.5221 to 0.2912 | 0.5572 | 0.5775 | ns |
| Comp.7 | -0.1142 | 0.2113 | -0.5288 to 0.3004 | 0.5406 | 0.5889 | ns |
| Comp.8 | 0.2447 | 0.2080 | -0.1635 to 0.6529 | 1.176 | 0.2397 | ns |
| Comp.9 | 0.4893 | 0.2372 | 0.02385 to 0.9547 | 2.063 | 0.0394 | * |
| Comp.10 | -0.7089 | 0.2147 | -1.130 to -0.2877 | 3.302 | 0.0010 | *** |
| Stimulants | -0.001639 | 0.02533 | -0.05135 to 0.04807 | 0.06470 | 0.9484 | ns |
| Cocaine | 0.04852 | 0.03068 | -0.01168 to 0.1087 | 1.581 | 0.1141 | ns |
| Narcotics | -0.007934 | 0.03272 | -0.07213 to 0.05626 | 0.2425 | 0.8084 | ns |
| Hallucinogens | 0.03203 | 0.03092 | -0.02865 to 0.09270 | 1.036 | 0.3006 | ns |
| Phencyclidine | -0.1380 | 0.04474 | -0.2258 to -0.05026 | 3.086 | 0.0021 | ** |
| Inhalants | -0.03072 | 0.03773 | -0.1047 to 0.04330 | 0.8142 | 0.4157 | ns |
| Cannabis | -0.01380 | 0.01816 | -0.04943 to 0.02183 | 0.7601 | 0.4473 | ns |
| Tranquilizers | 0.02404 | 0.02963 | -0.03410 to 0.08219 | 0.8114 | 0.4173 | ns |
| Alcohol | -0.0005173 | 0.002678 | -0.005771 to 0.004737 | 0.1932 | 0.8469 | ns |

1. **The effect of substance use disorders on the association of *SENP7**cg09657378 and lifetime PTSD**

| **Variable** | **Estimate** | **Standard error** | **95% CI (asymptotic)** | **\|t\|** | **P value** | **P value summary** |
| --- | --- | --- | --- | --- | --- | --- |
| Intercept | -2.792 | 0.6912 | -4.148 to -1.436 | 4.040 | <0.0001 | **** |
| LT PTSD | -0.1375 | 0.02764 | -0.1917 to -0.08330 | 4.976 | <0.0001 | **** |
| Age | -0.002447 | 0.0005635 | -0.003552 to -0.001341 | 4.342 | <0.0001 | **** |
| Smoking | 0.02444 | 0.02003 | -0.01487 to 0.06375 | 1.220 | 0.2227 | ns |
| CD34 | -2.711 | 0.5878 | -3.864 to -1.558 | 4.612 | <0.0001 | **** |
| CD14 | -1.652 | 0.4698 | -2.574 to -0.7304 | 3.517 | 0.0005 | *** |
| Buccal | -2.273 | 0.5470 | -3.346 to -1.200 | 4.155 | <0.0001 | **** |
| PC1 | 15.10 | 12.85 | -10.11 to 40.32 | 1.175 | 0.2402 | ns |
| PC2 | -3.687 | 1.151 | -5.946 to -1.428 | 3.203 | 0.0014 | ** |
| PC3 | -0.1635 | 0.2410 | -0.6363 to 0.3094 | 0.6784 | 0.4977 | ns |
| PC4 | -0.08041 | 0.2694 | -0.6089 to 0.4481 | 0.2985 | 0.7654 | ns |
| PC5 | 0.3781 | 0.2110 | -0.03600 to 0.7921 | 1.792 | 0.0735 | ns |
| PC6 | -0.1186 | 0.2066 | -0.5240 to 0.2868 | 0.5739 | 0.5662 | ns |
| PC7 | -0.1110 | 0.2113 | -0.5255 to 0.3036 | 0.5251 | 0.5996 | ns |
| PC8 | 0.2104 | 0.2076 | -0.1970 to 0.6178 | 1.013 | 0.3111 | ns |
| PC9 | 0.4738 | 0.2364 | 0.009903 to 0.9378 | 2.004 | 0.0453 | * |
| PC10 | -0.6912 | 0.2145 | -1.112 to -0.2702 | 3.222 | 0.0013 | ** |
| Substance use disorder | 0.01306 | 0.01963 | -0.02545 to 0.05157 | 0.6656 | 0.5058 | ns |
| Alcohol use disorder | -0.01294 | 0.01238 | -0.03722 to 0.01134 | 1.045 | 0.2961 | ns |
| Tobacco use disorder | -0.02280 | 0.01593 | -0.05405 to 0.008447 | 1.432 | 0.1525 | ns |

**Supplementary Figure Legends**

**Supplementary Figure 1. Quantile-Quantile Plots.**

Quantile-quantile plot illustrates the *p*-values of the association between DNA methylation and PTSD. There is no evidence for inflation (λ=1.006 for current PTSD, and 1.02 for lifetime PTSD).

**Supplementary Figure 2. Violin Plots of Genome-Wide Significant (GWS) CpG Sites Associated with PTSD.**

DNA methylation levels (beta values) of GWS CpG sites associated with **A)** current and **B)** lifetime PTSD are shown.

**Supplementary Figure 3. Methylation Quantitative Trait Loci (meQTL) of Genome-Wide Significant CpG Sites Associated with PTSD.**

A manhattan plot is shown with the meQTLs of PTSD-associated GWS CpG sites. Significance threshold is set at 4.0 x 10^-6^.

**Supplementary Figure 1. Quantile-Quantile plots.**

**A)**

**
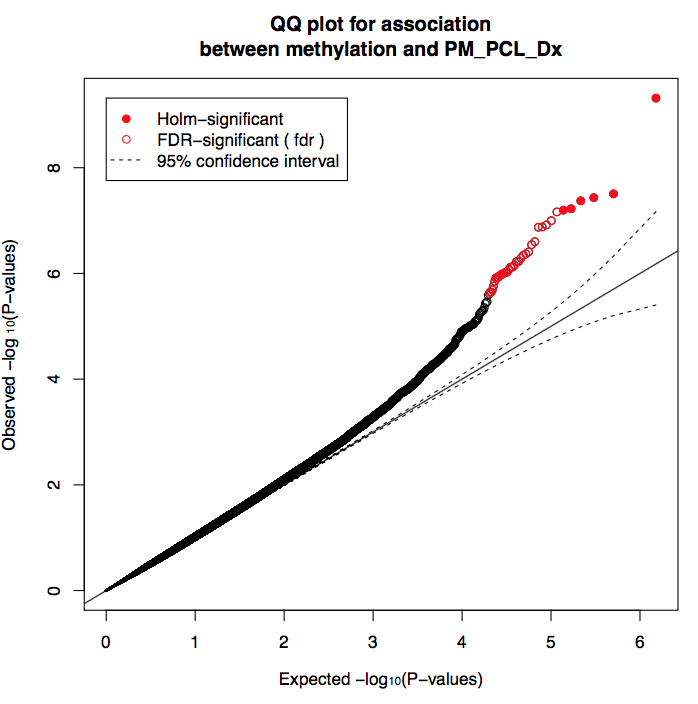
**

**B)**

**
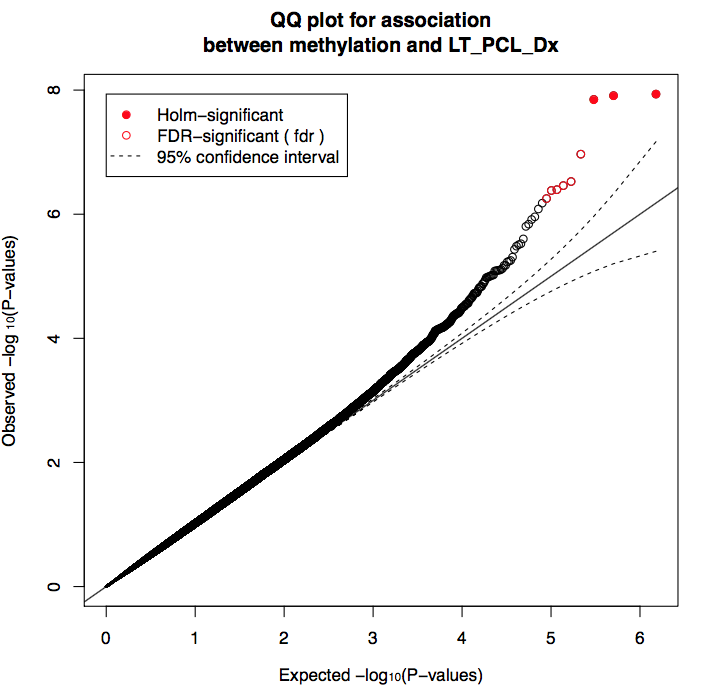
**

**Supplementary Figure 2. Violin Plots of Genome-Wide Significant (GWS) CpG Sites Associated with PTSD.**

**A) Current PTSD**

**1. 2. 3.**

**
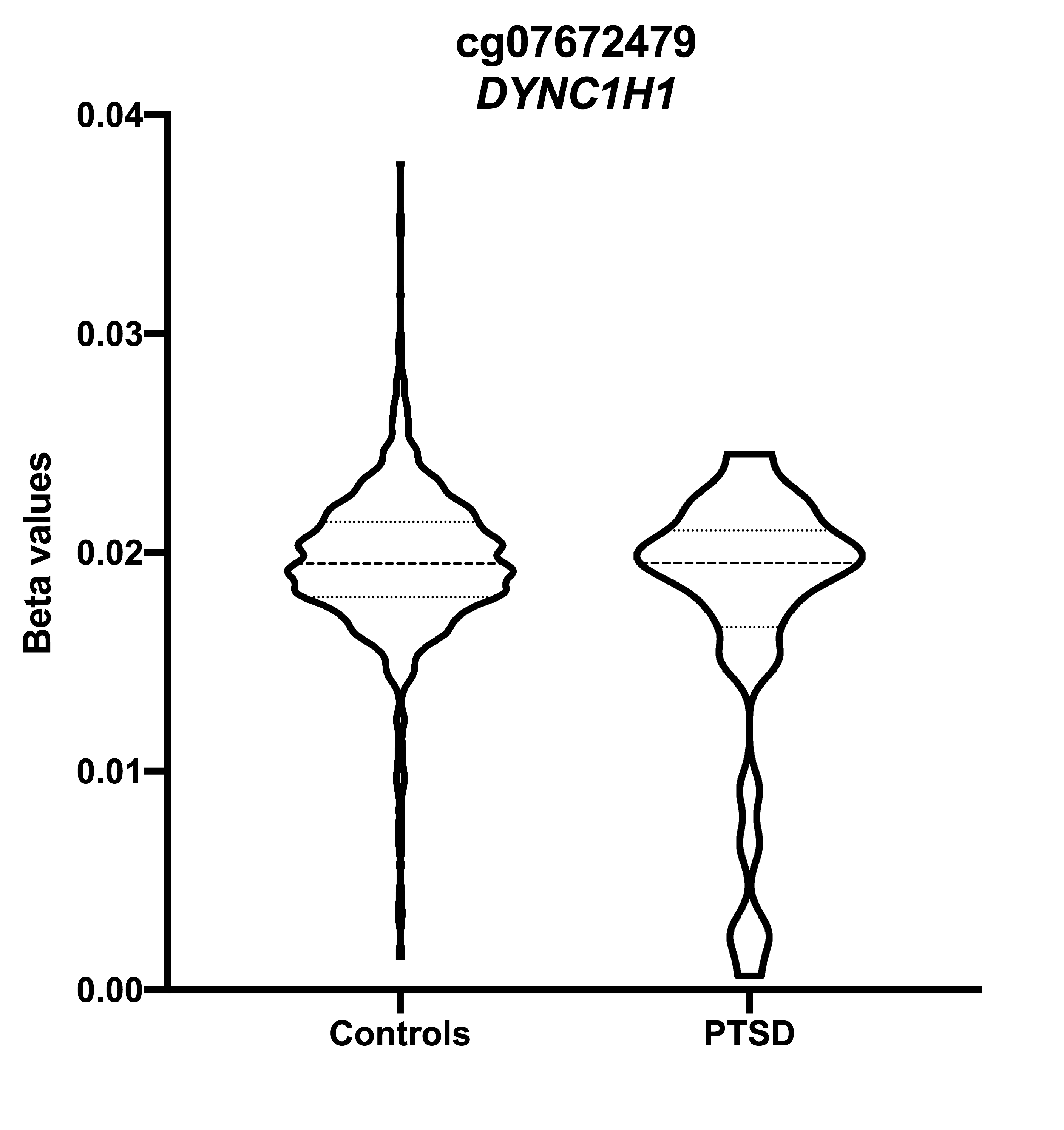

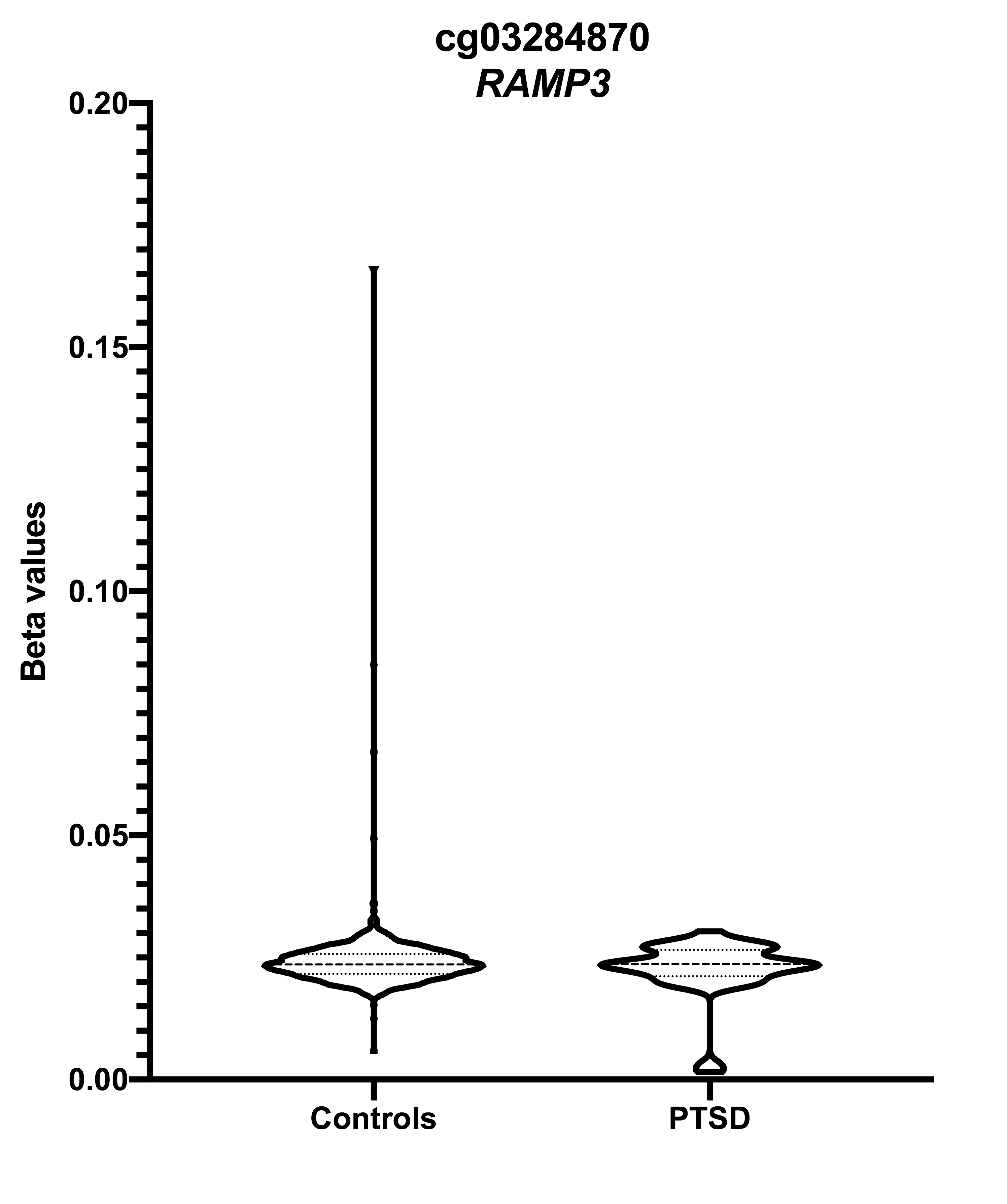

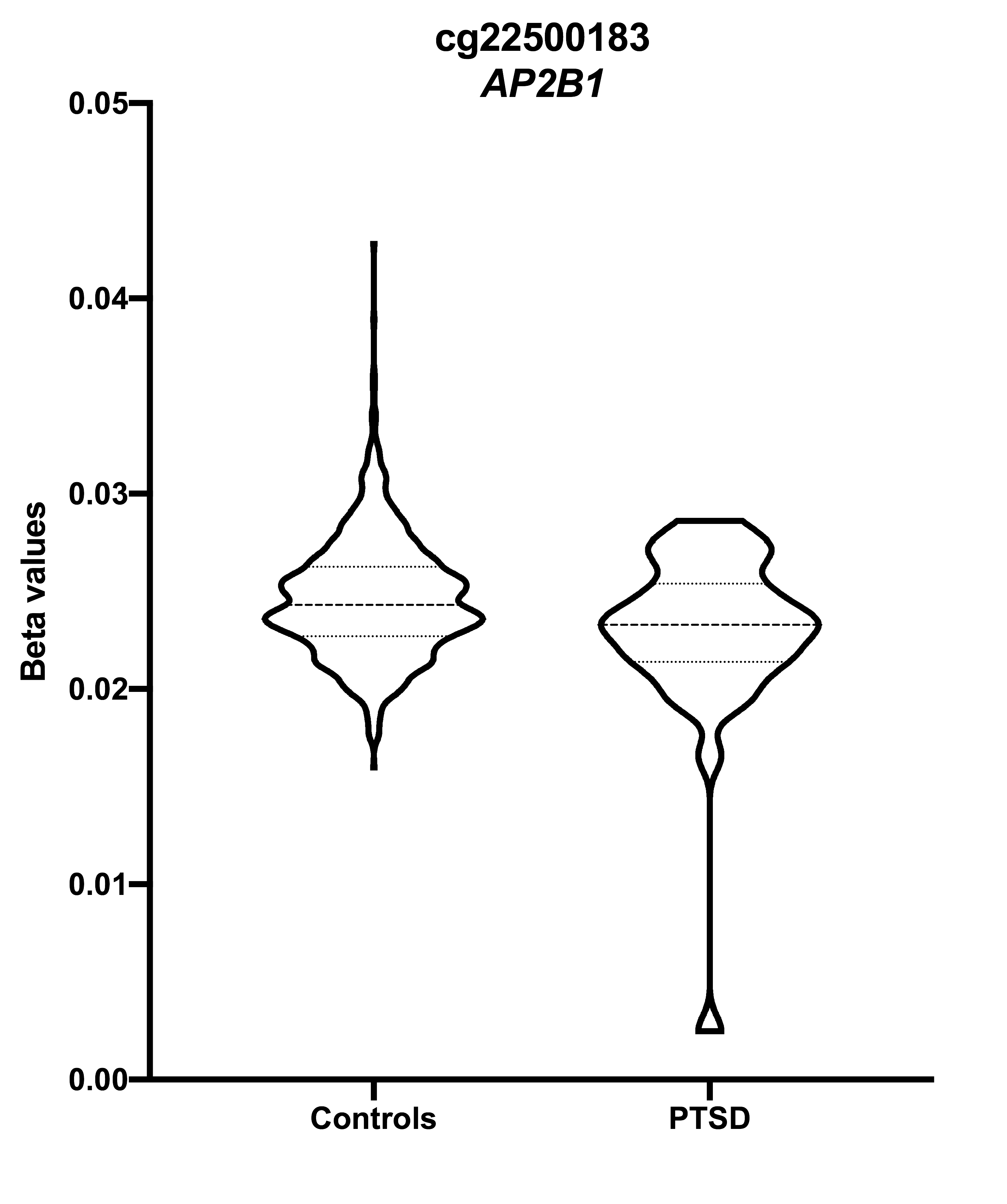
**

**4. 5. 6.**

**
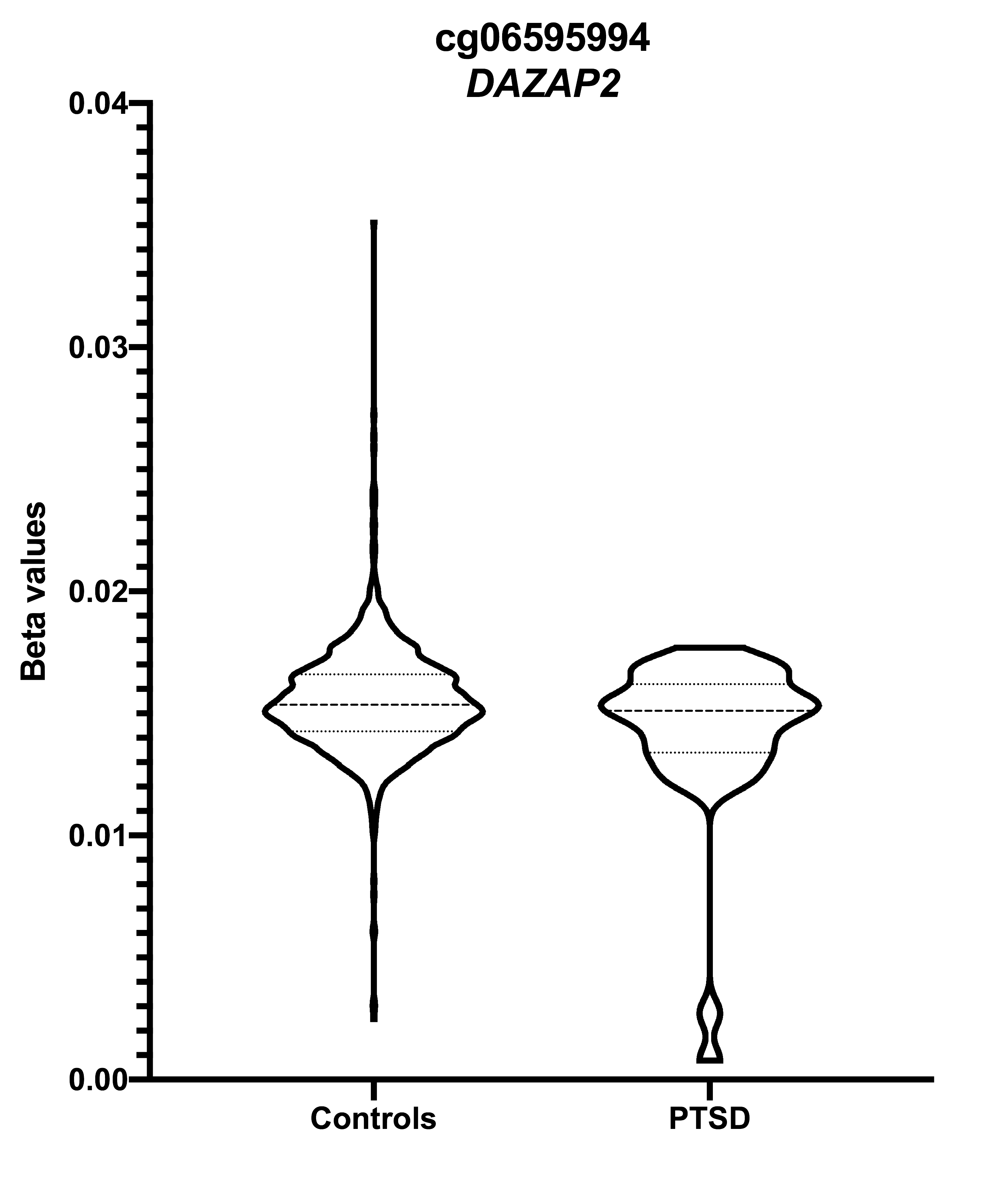

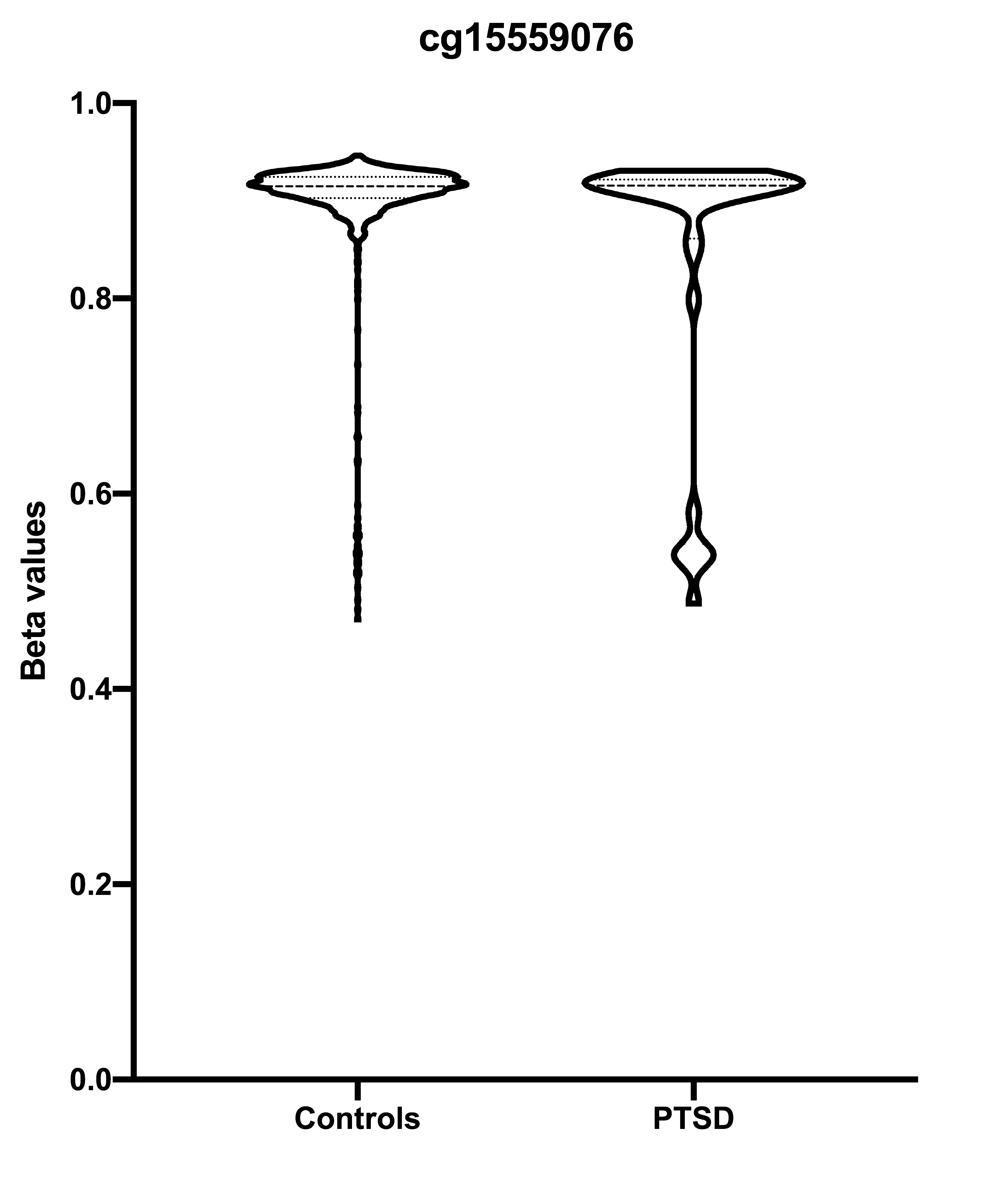

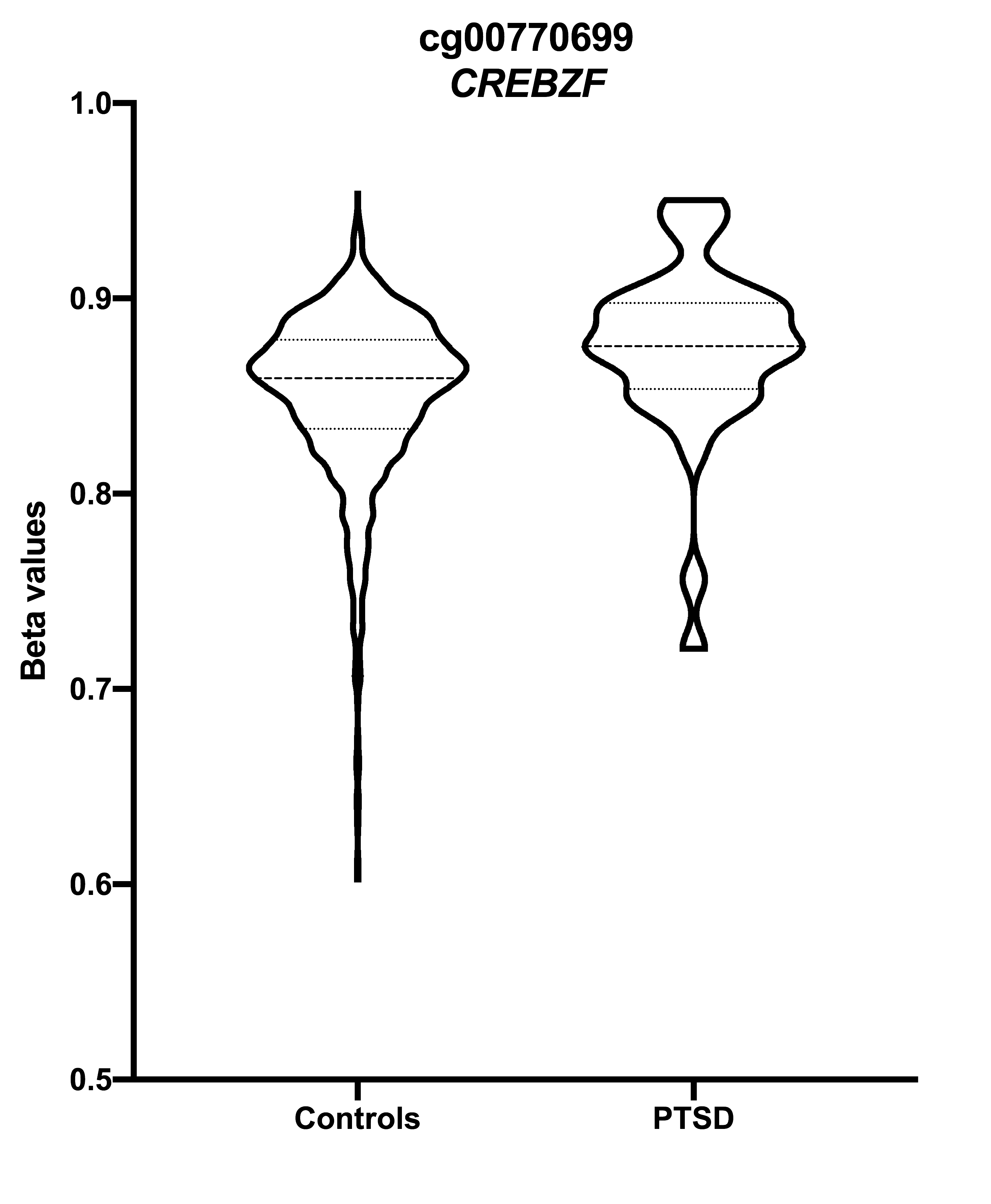
**

**B) Lifetime PTSD**

**1. 2. 3.**

**
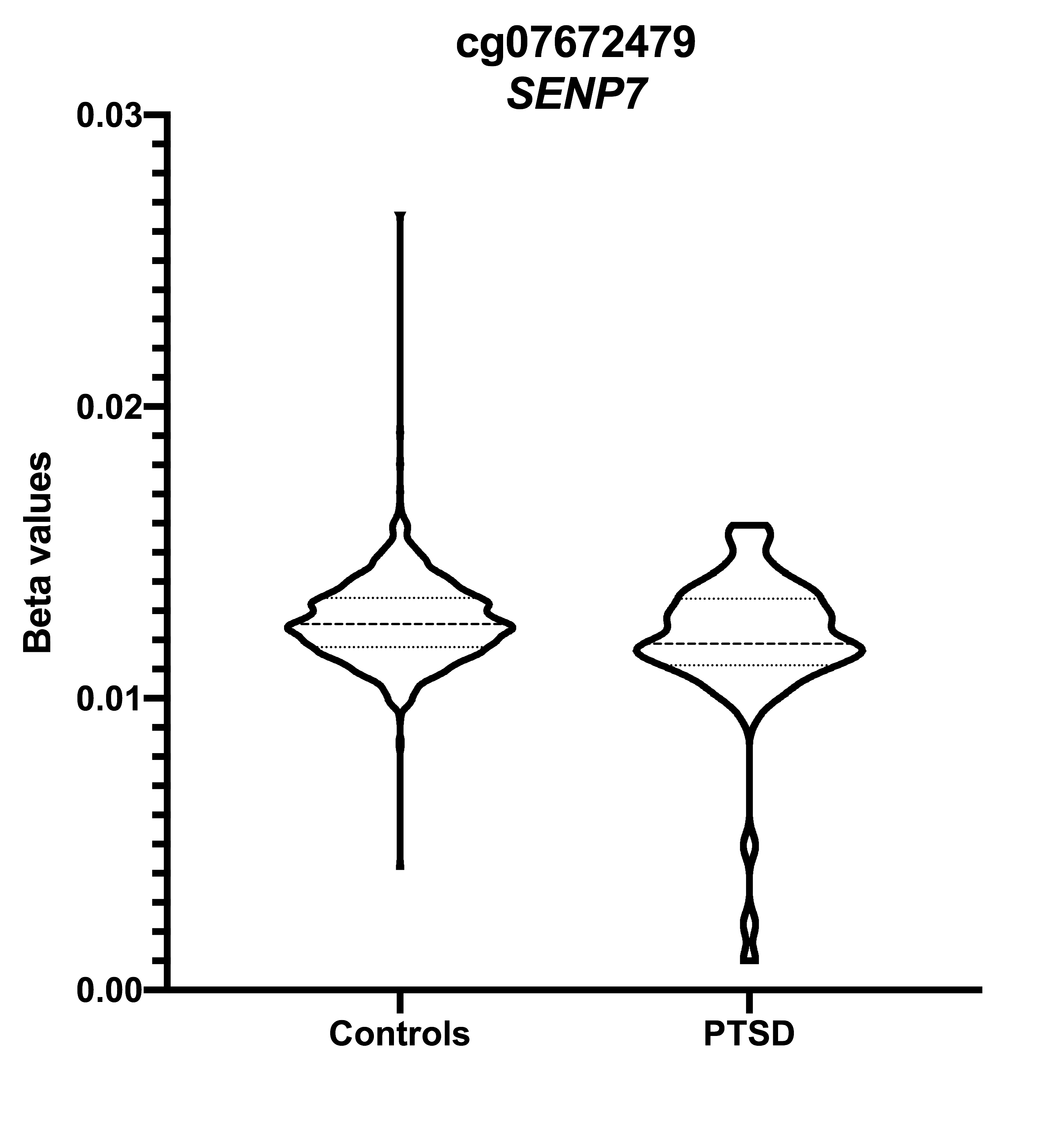

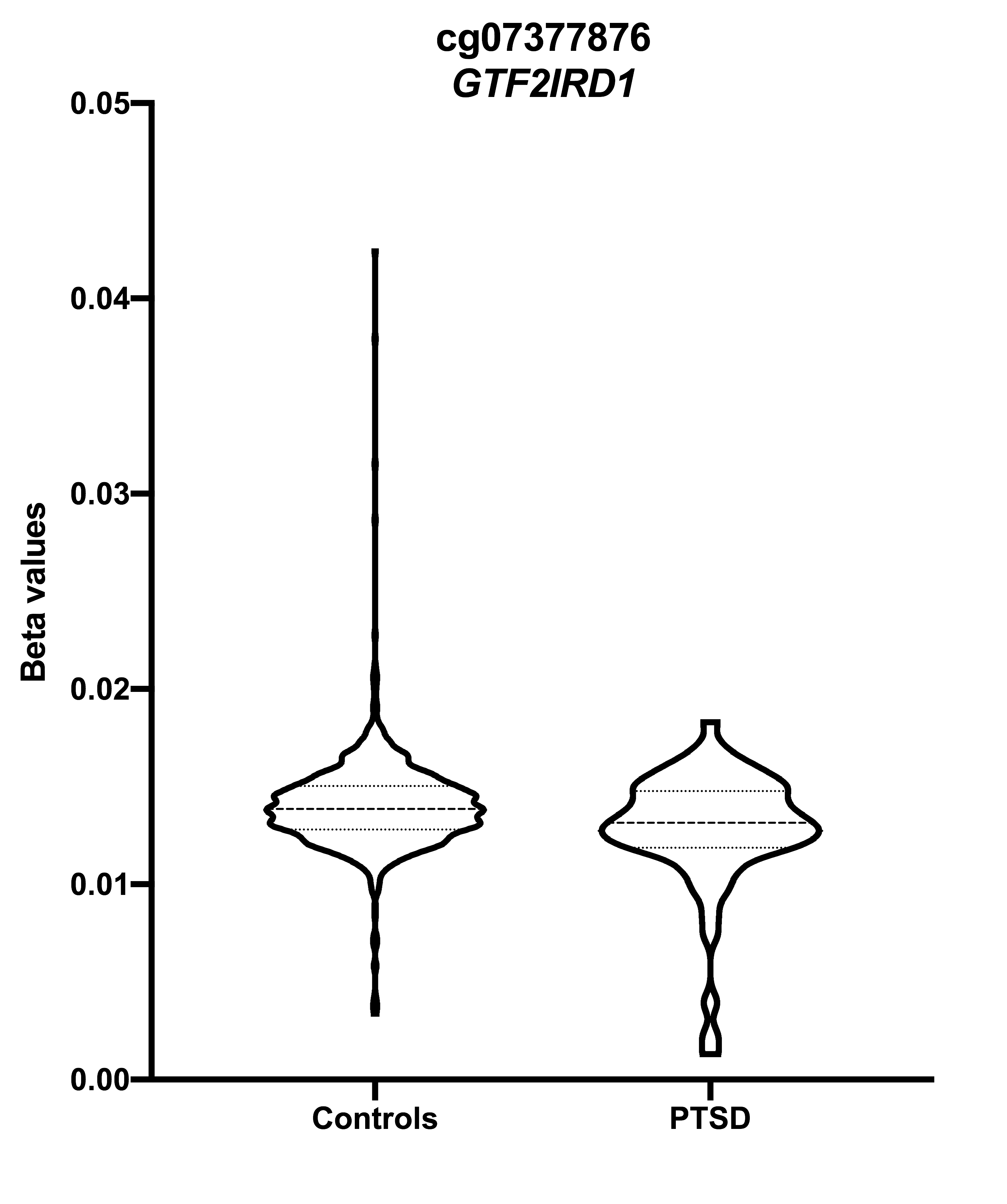

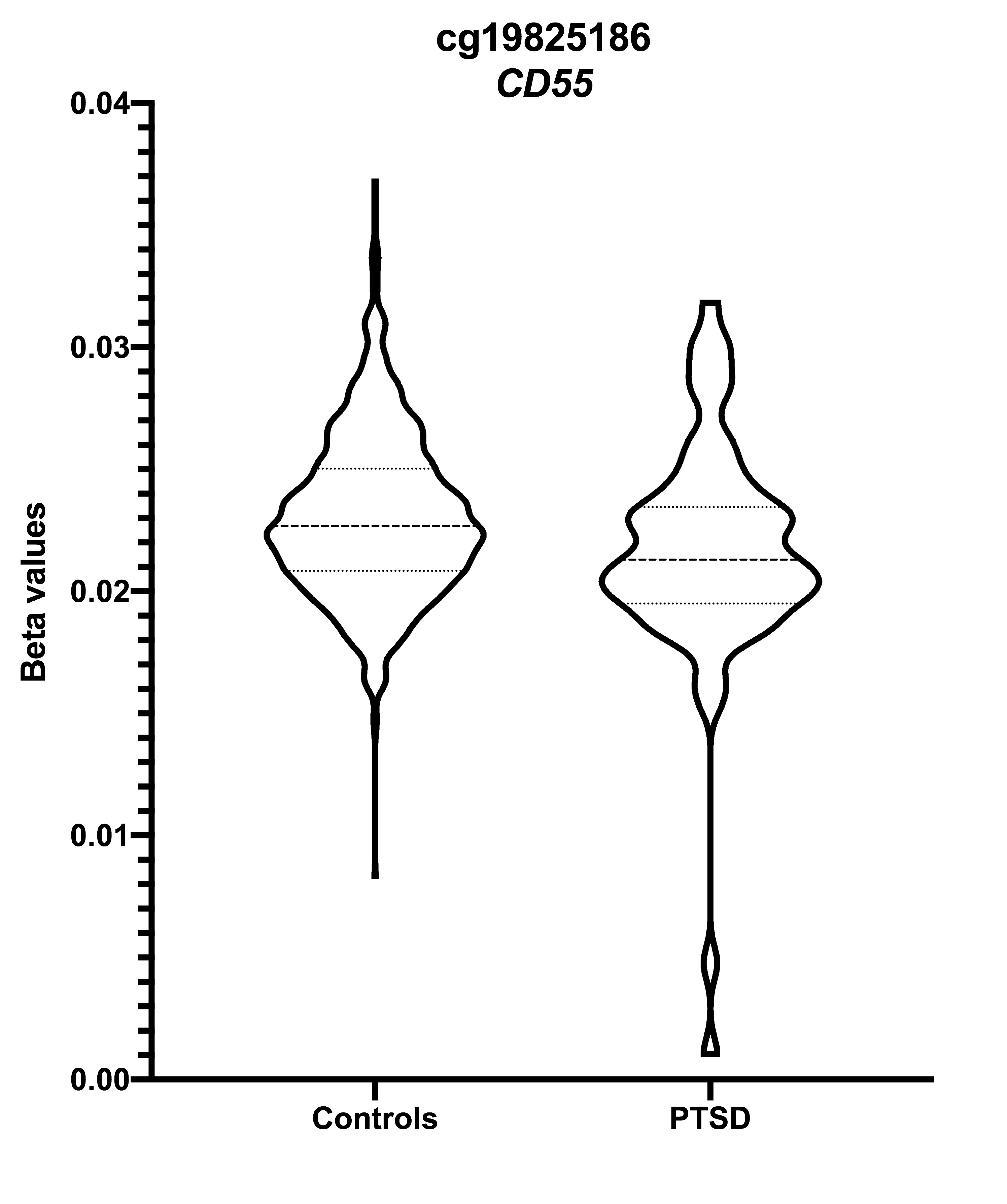
**

**Supplementary Figure 3. Methylation Quantitative Trait Loci
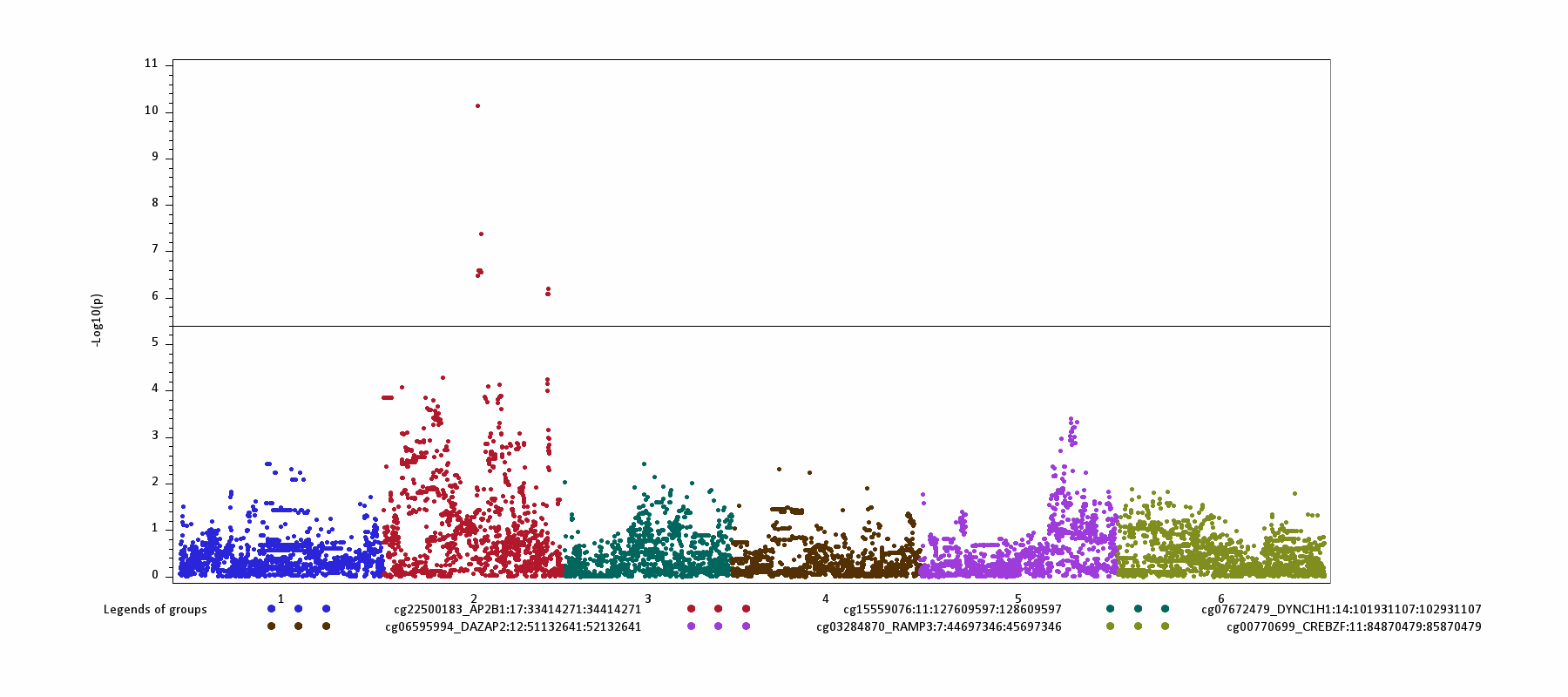
**
